# Identifying intrinsic and extrinsic determinants that regulate internal initiation of translation mediated by the FMR1 5' leader

**DOI:** 10.1186/1471-2199-9-89

**Published:** 2008-10-15

**Authors:** Tara Dobson, Erika Kube, Stephanie Timmerman, Les A Krushel

**Affiliations:** 1 Department of Pharmacology, University of Colorado Denver School of Medicine, Aurora, CO 80045, USA; 2 Department of Biochemistry and Molecular Genetics, University of Colorado Denver School of Medicine, Aurora, CO 80045, USA; 3 Neurosciences Program, University of Colorado Denver School of Medicine, Aurora, CO 80045, USA

## Abstract

**Background:**

Regulating synthesis of the Fragile X gene (FMR1) product, FMRP alters neural plasticity potentially through its role in the microRNA pathway. Cap-dependent translation of the FMR1 mRNA, a process requiring ribosomal scanning through the 5' leader, is likely impeded by the extensive secondary structure generated by the high guanosine/cytosine nucleotide content including the CGG triplet nucleotide repeats in the 5' leader. An alternative mechanism to initiate translation – internal initiation often utilizes secondary structure to recruit the translational machinery. Consequently, studies were undertaken to confirm and extend a previous observation that the FMR1 5' leader contains an internal ribosomal entry site (IRES).

**Results:**

Cellular transfection of a dicistronic DNA construct containing the FMR1 5' leader inserted into the intercistronic region yielded significant translation of the second cistron, but the FMR1 5' leader was also found to contain a cryptic promoter possibly confounding interpretation of these results. However, transfection of dicistronic and monocistronic RNA *ex vivo *or *in vitro *confirmed that the FMR1 5' leader contains an IRES. Moreover, inhibiting cap-dependent translation *ex vivo *did not affect the expression level of endogenous FMRP indicating a role for IRES-dependent translation of FMR1 mRNA. Analysis of the FMR1 5' leader revealed that the CGG repeats and the 5' end of the leader were vital for internal initiation. Functionally, exposure to potassium chloride or intracellular acidification and addition of polyinosinic:polycytidylic acid as mimics of neural activity and double stranded RNA, respectively, differentially affected FMR1 IRES activity.

**Conclusion:**

Our results indicate that multiple stimuli influence IRES-dependent translation of the FMR1 mRNA and suggest a functional role for the CGG nucleotide repeats.

## Background

The mRNA and protein generated from the FMR1 gene in neurons is localized to dendrites [1,2]. The FMR1 protein, FMRP is synthesized in response to neural activity and its function as an RNA binding protein influences the translational level of other dendritically localized mRNAs [3-6]. FMRP is also part of the RISC complex [7,8], a set of proteins that interact with micro-RNAs or short interfering RNAs to inhibit translation and or degrade the RNA, respectively.

Regulating the synthesis of FMRP is important for cellular function. FMRP over-expression leads to a defect in dendritic architecture, synaptic differentiation, and abnormal behaviors in mice and flies [9,10]. Alternatively, the absence of FMRP in Fragile X Syndrome (FXS) leads to alterations in synaptic plasticity resulting in mental retardation [11]. FXS develops from an expansion of the CGG nucleotide repeats in the 5' leader of the FMR1 gene [12-14]. Normal individuals carry from 5 – 50 repeats while those with FXS carry over 200 repeats. The expansion increases methylation of the gene inhibiting transcription. In some cases transcription of the gene occurs [15,16], but translation of the mRNA is inhibited by the presence of the CGG repeat expansion [17]. The CGG repeats are evolutionarily conserved in mammals suggesting that the repeats have some function aside from inhibiting transcription and translation [18].

During post-transcriptional gene processing, a 7-methyl guanosine (termed the cap structure) is positioned at the 5' end of an mRNA [19]. All mRNAs can potentially be translated by the eukaryotic initiation factor (eIF) 4E binding to the cap structure and recruiting the remainder of the translational machinery including the ribosome [20]. On the other hand, a subset of mRNAs initiate translation in a cap-independent manner through internal ribosomal entry sites (IRESes) situated in the 5' leader and in some cases in the open reading frame [21,22]. IRES-dependent translation is thought to be utilized when cap-dependent translation is inhibited. This occurs during normal physiological processes including mitosis, but also in response to stressful events such as apoptosis [23-25]. It may also be a primary translational mechanism that can be regulated independently of pathways affecting cap-dependent translation. In the nervous system, numerous dendritically localized mRNAs contain IRESes including those coding for the alpha subunit of CAMKII, activity-related cytoskeletal protein, and the neurotrophin receptor TrkB [26,27]. The high preponderance of dendritically localized mRNAs containing IRESes suggests that IRES-dependent translation is an important protein synthesis mechanism in dendrites.

Our goals were to confirm a previous study that the FMR1 5' leader mediates internal initiation of translation [28], identify regions in the FMR1 5' leader critical for IRES activity, and to determine if FMR1 IRES activity is affected by cellular processes in which FMRP participates. Initially, we re-examined the FMR1 5' leader for IRES activity and found that it contained a cryptic promoter, compelling the use of RNA constructs. Translation assays using RNA both *in vitro *and *ex vivo *demonstrated that the FMR1 5' leader does contain an IRES and that IRES-dependent translation may be an important mechanism for the synthesis of FMRP *in vivo*. A dissection of the 5' leader showed that the 5' 45 nucleotides (nt) as well as the CGG repeats are important for internal initiation. Finally, multiple cellular stimuli including exposure to KCl and intracellular acidification as models for neural activity and exposure to polyinosinic:polycytidylic acid as a model for the presence of double stranded RNA resulted in alterations of FMR1 IRES activity.

## Results

### The FMR1 5' leader directs expression of the second cistron in a dicistronic DNA construct

**Figure 1:**
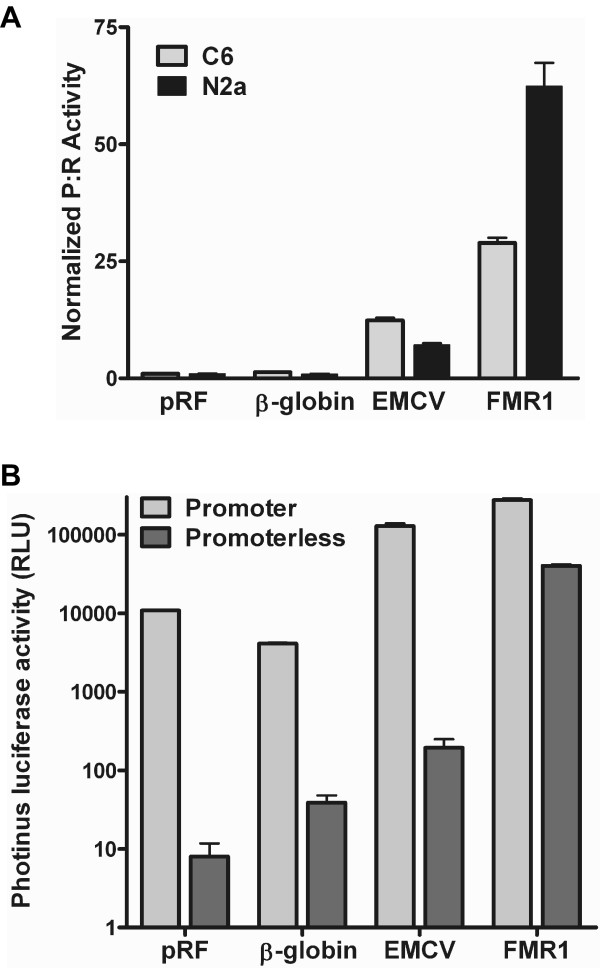
**The FMR1 5' leader exhibits putative IRES-activity, but also contains a cyrptic promoter.** (A) Dicistronic luciferase DNA constructs containing the multiple cloning site (MCS) from pRF, the 5' leader from the β-globin or FMR1, or the EMCV IRES inserted into the intercistronic region were transfected individually into C6 cells. Luciferase activity is shown as the ratio of *Photinus *luciferase to *Renilla *luciferase (P:R) and is normalized to the activity obtained from the control construct, pRF. A P:R ratio that is above that obtained from pRF indicates the presence of an IRES. (B) Dicistronic luciferase DNA constructs containing the MCS from pRF, the β-globin or FMR1 5' leader or the EMCV IRES with or without the SV40 promoter were transfected into C6 cells. The *Photinus *luciferase activity from each transfection is shown. Each experiment was performed in triplicate, n = 3.

Viral IRESes are denoted by being relatively long (> 200 nt), guanosine/cytosine (G/C) nt rich, and containing upstream open reading frames (uORFs). The FMR1 5' leader exhibits a subset of these characteristics being approximately 240 nt (depending upon the number of CGG repeats) and is > 80% G/C rich, but it does not contain any uORFs. To confirm a previous report that the FMR1 5' leader contained an IRES [28], the leader was inserted into the intercistronic region of a dicistronic luciferase construct. Two negative controls were used, the multiple cloning site (MCS) in the intercistronic region (pRF) and the β-globin 5' leader. The β-globin 5' leader was chosen since the β-globin mRNA is translated exclusively in a cap-dependent manner [29]. The encephalomyocarditis (EMCV) virus IRES was chosen as the positive control [30]. The constructs were transfected into the C6 glioma and N2a neuroblastoma cell lines. After 24 hrs the cells were harvested and assayed for *Renilla *and *Photinus *luciferase. The *Photinus*:*Renilla *luciferase (P:R) ratio obtained from the control dicistronic construct (pRF) was normalized to one. A ratio above one would indicate IRES activity. Both the EMCV IRES and the FMR1 5' leader exhibited P:R ratios significantly higher than that observed from the pRF (and β-globin) construct. Indeed, the P:R ratio obtained from the FMR1 5' leader was approximately 30 and 60 fold higher in C6 and N2a cells respectively (Fig. 1A). The level of FMR1 IRES activity was higher than that observed from the EMCV IRES. This initial result suggests that the FMR1 5' leader may contain an IRES.

### The FMR1 5' leader contains a cryptic promoter

In addition to internal initiation, increased levels of *Photinus *luciferase protein can be generated from the dicistronic luciferase constructs through cryptic splicing or cryptic promoter activity. For example, the presence of a cryptic promoter in the 5' leader will lead to the production of a monocistronic *Photinus *luciferase mRNA [31,32] and artificially increase the P:R ratio. To determine if cryptic promoter activity was present in the FMR1 5' leader, the dicistronic luciferase constructs with or without the SV40 promoter and intron were transfected into C6 cells. *Photinus *luciferase activity from the promoterless constructs containing the pRF MCS, β-globin 5' leader, and EMCV IRES was very low, less than 1% of the *Photinus *luciferase activity obtained from the constructs with the intact promoter. This result confirms previous studies indicating that these leaders do not contain a cryptic promoter [26,33]. However, the promoterless construct containing the FMR1 5' leader generated approximately 15% of the 'total' *Photinus *luciferase activity. After subtracting the minor contribution of the cryptic promoter to the translation of the second cistron, the data still suggests that the FMR1 5' leader has an IRES, but it does temper this conclusion.

### The FMR1 5' leader exhibits IRES activity from a dicistronic RNA

**Figure 2:**
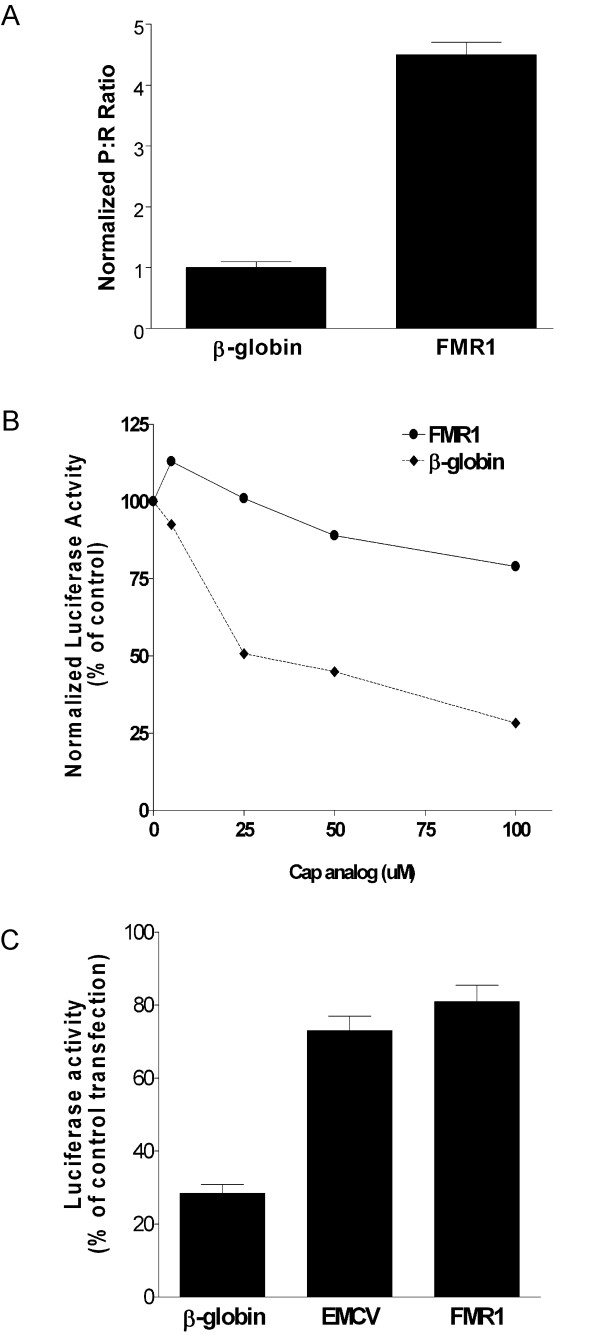
***Ex vivo *****and *****in vitro***** studies demonstrating IRES activity mediated by the FMR1 5' leader**. (A) Capped dicistronic luciferase RNA containing the 5' leader from the β-globin or FMR1 mRNA inserted into the intercistronic region was transfected individually into C6 cells. Luciferase activity is shown as the ratio of *Photinus *luciferase to *Renilla *luciferase (P:R) and is normalized to the activity of the β-globin construct. The experiment was performed in triplicate, n = 3. (B) Monocistronic *Photinus *luciferase mRNA containing the β-globin or FMR1 5' leader was translated in rabbit reticulocyte lysate in the presence of increasing concentrations of cap analog. The initial level of *Photinus *luciferase activity from each monocistronic mRNA was normalized to 100. (C) Monocistronic *Photinus *luciferase constructs containing the β-globin, EMCV, or FMR1 5' leader were co-transfected with either a plasmid expressing hypophosphorylated 4E-BP1 or a control plasmid and assayed for luciferase activity. The activities obtained in cells co-transfected with 4E-BP1 is represented as a percentage of the activity obtained in cells co-transfected with the control plasmid. The experiment was performed in triplicate, n = 3.

To determine more unambiguously whether the FMR1 5' leader contains an IRES, the dicistronic constructs were transcribed *in vitro *eliminating the possibility of alternative splicing and alternate promoters. Transfecting the mRNA into C6 cells did provide evidence of IRES activity (Fig. 2A). The P:R ratio obtained from the dicistronic luciferase mRNA containing the FMR1 5' leader was approximately 4.5 fold higher than the β-globin control. Although the P:R ratio generated from the FMR1 5' leader was substantially lower when comparing the RNA to DNA transfections, the results still indicate that the FMR1 5' leader contains an IRES.

### Translation of a monocistronic mRNA *in vitro *and *ex vivo *indicates a key role for IRES-dependent translation mediated by the FMR1 5' leader

The dicistronic luciferase assay is useful to identify sequences that can internally initiate translation, but it does not indicate the role of an IRES in a monocistronic mRNA, the context in which the IRES is normally found in cellular mRNA. Consequently, two approaches were utilized to determine whether the FMR1 IRES is a major contributor to the translation of a monocistronic capped mRNA. Initially, *in vitro *transcribed monocistronic mRNA containing the *Photinus *luciferase open reading frame (ORF) and the β-globin or FMR1 5' leader was translated in rabbit reticulocyte lysate. The overall level of *Photinus *luciferase synthesis was reduced by approximately 40% when the FMR1 5' leader was present. This result is not surprising since cap-dependent translation of a short unstructured 5' leader (β-globin) is very efficient. Increasing concentrations of cap analog were added to the lysate to compete with the cap structure for eIF-4E and inhibit cap-dependent translation. Translation of the mRNA containing the β-globin 5' leader decreased as the concentration of cap analog increased (Fig. 2B). This result demonstrates that the β-globin mRNA is being translated in a cap-dependent manner. On the other hand, translation of the mRNA containing the FMR1 5' leader was only moderately affected (Fig. 2B); translation of the *Photinus *luciferase cistron decreased by only 15% at the highest concentration of cap analog. This result not only indicates that the mRNA containing the FMR1 5' leader is being translated in a cap-independent manner, but that it may be the major mechanism for its translation.

To determine the role of the FMR1 IRES within a cell, cap-dependent translation was inhibited *ex-vivo*. The 4E-Binding Protein 1 (4E-BP1) binds and sequesters eIF-4E preventing cap-dependent translation [34], but phosphorylation of 4E-BP1 decreases its affinity to eIF-4E. Consequently, C6 cells were transfected with a construct coding for a mutant of 4E-BP1 (4E-BPmut) with the two key phosphorylation sites mutated (Thr – 37 – Ala/Thr – 46 – Ala) or a control plasmid [20]. Monocistronic constructs containing the β-globin, EMCV, or FMR1 5' leader were co-transfected. In the presence of over-expressed 4E-BPmut, the level of *Photinus *luciferase activity derived from the mRNA containing the β-globin 5' leader decreased by 72% (Fig. 2C). However, translation from the mRNAs containing the FMR1 or EMCV 5' leader only decreased by 19% and 27%, respectively (Fig. 2C). Since 81% of the luciferase activity remains when cap-dependent translation is inhibited, it indicates that IRES-dependent translation may be the primary mechanism for translation of the FMR1 mRNA *in vivo*.

### Endogenous FMRP expression is unaffected by reducing cap-dependent translation

**Figure 3:**
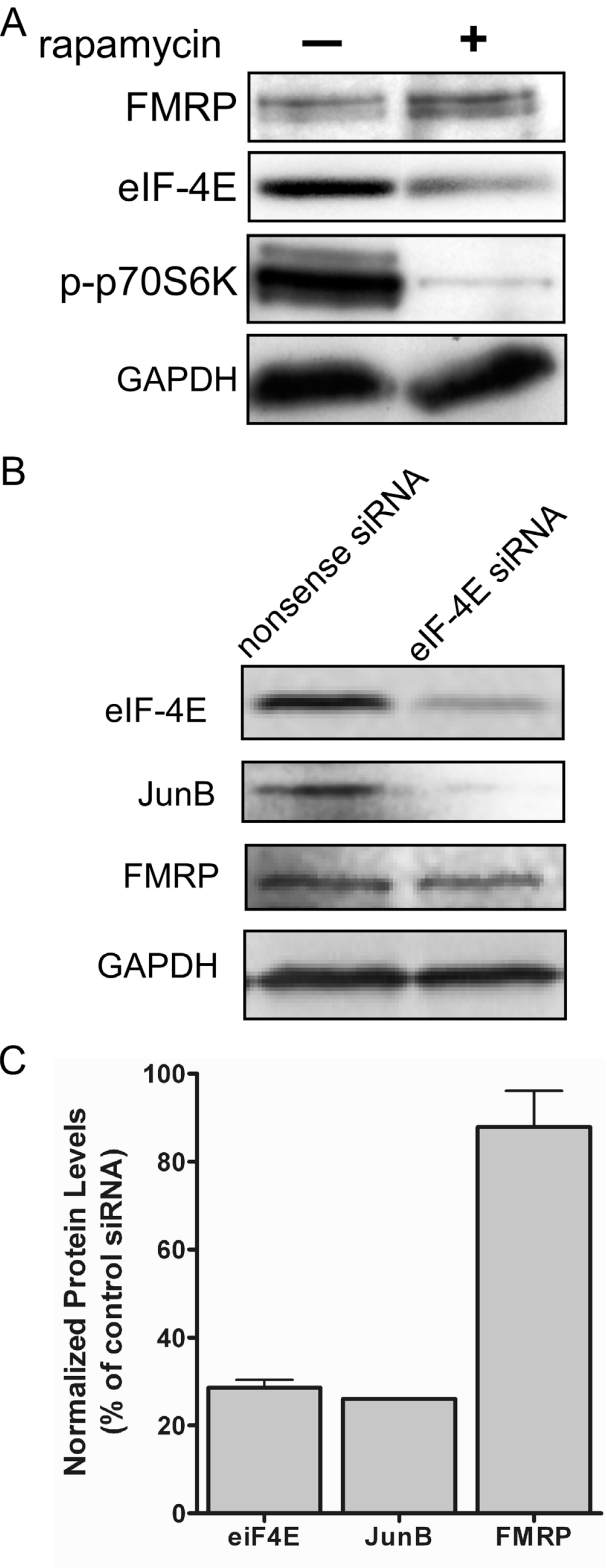
**FMRP expression is maintained when cap-dependent translation is reduced by rapamycin or eIF-4E siRNA.(A) **Lysates from untreated or rapamycin treated C6 cells were analyzed for FMRP, phosphorylated p70 S6 kinase, and eIF-4E. Shown is a representative Western blot for each experiment, n = 3. (B) siRNA directed against eIF-4E or a nonsense siRNA were transfected into C6 cells for 72 hr. Lysates from these cells were analyzed for eIF-4E, FMRP, JunB and GAPDH. GAPDH was used as a loading control since it has an extended half-life. Shown is a representative Western blot for each experiment, n = 3. (C) Western blots of lysates from cells treated with eIF-4E or a nonsense siRNA. Expression level of the proteins were quantitated using ImageQuant software (n = 3) and were normalized to GAPDH levels.

To examine whether IRES-dependent translation is utilized for the synthesis of endogenous FMRP, cap-dependent translation was inhibited by treating C6 cells with rapamycin. The mammalian target of rapamycin (mTOR) is a kinase that is a key link in the promotion of cap-dependent translation by phosphorylating 4E-BP and p70 S6 kinase. Exposure to 20 nM rapamycin for 24 hrs actually led to a modest increase in FMRP expression (Fig. 3A). Phosphorylation of p70 S6 kinase was abolished demonstrating that the mTOR pathway was inhibited and the expression level of eIF-4E was decreased indicating cap-dependent translation was repressed (Fig. 3A). These results indicate that FMRP can be expressed under conditions in which cap-dependent translation is impeded.

In addition to inhibiting cap-dependent translation, blocking mTOR activity can alter other cellular pathways and indirectly alter translation. To more directly examine whether IRES-dependent translation is utilized for the synthesis of endogenous FMRP, cap-dependent translation was inhibited by knocking-down expression of eIF-4E. C6 cells were exposed to a pool of siRNA (Dharmacon) directed against the eIF-4E mRNA or a nonsense siRNA for 72 hr. Quantitation of Western blots showed that eIF-4E expression was reduced by > 70% (Fig. 3B, C). The level of the transcription factor JunB whose mRNA is translated in a cap-dependent manner was also reduced by > 75%. However, FMRP expression was decreased by only 12% (Fig. 3B, C). The observations that FMRP expression is maintained despite reduced levels of eIF-4E or mTOR activity (detailed above) indicates that IRES-dependent translation is likely a important contributor to the synthesis of FMRP.

### Multiple regions in the 5' leader contribute to FMR1 IRES activity

**Figure 4:**
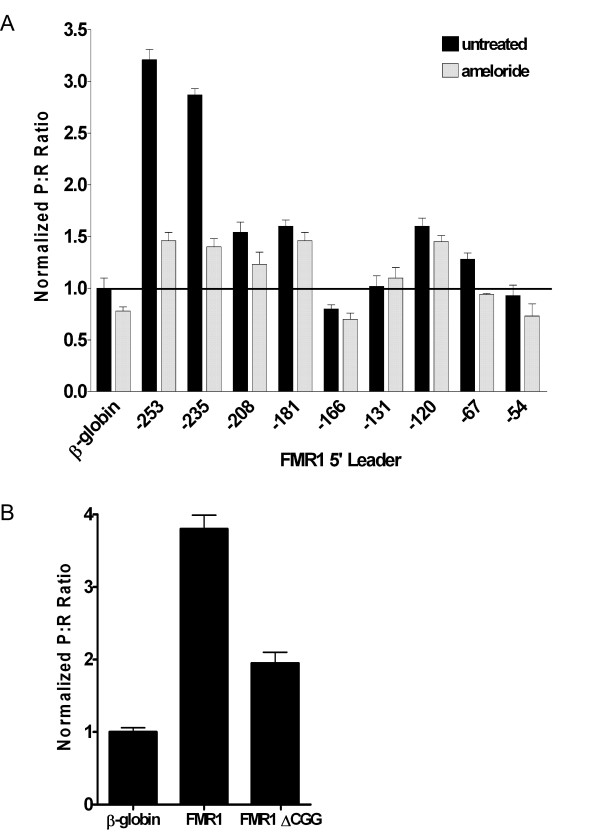
**Truncations and ameloride treatment identify regions in the FMR1 5' leader important for IRES activity.** (A) Dicistronic luciferase mRNA containing the β-globin, FMR1, or serial 5' truncations of the FMR1 5' leader inserted into the intercistronic region were transfected into C6 cells. C6 cells were exposed to ameloride for 24 hr or untreated. Luciferase activity is shown as the P:R ratio and is normalized to the activity from the β-globin mRNA. (B) Dicistronic luciferase mRNA containing the full length FMR1 5' leader, FMR1 5' leader with an internal deletion of the CGG repeats (FMRΔCGG), or the β-globin 5' leader inserted into the intercistronic region was transfected into C6 cells. Luciferase activity is shown as the P:R ratio and is normalized to the activity from the β-globin mRNA. Each experiment was performed in triplicate, n = 3.

A deletional analysis was performed to identify regions in the FMR1 5' leader important for IRES activity. Serial 5' truncations ranging from 11 to 53 nt in length were created. The abridged 5' leaders were inserted into the dicistronic luciferase construct and *in vitro *transcribed mRNA was transfected into C6 cells. The initial truncations resulted in a loss of IRES activity. Truncating the nt from ^-^235 to ^-^208 resulted in the largest decrease in IRES activity with the resulting P:R ratio being only 1.5 fold over that obtained from the β-globin control (Fig. 4A). Deleting an additional 42 nt (^-^208 – ^-^167) abolished all IRES activity. Additional truncations of 46 nt (^-^166 – ^-^121) actually led to an increase in the P:R ratio of approximately 1.5 fold over the control value. Further truncations of 53 and 13 nt (^-^120 – ^-^54) that encompassed the CGG repeats resulted in a decrease and complete loss of IRES activity, respectively. These results indicate that 1) the 5' end of the 5' leader is important for wild-type IRES activity, 2) an internal region may be inhibitory, and 3) the region including ^-^120 – ^-^54 exhibits a basal level of IRES activity. Interestingly, this latter region encompasses the CGG repeats of which nine are contained in the present 5' leader.

### Changes in intracellular pH regulate the FMR1 IRES

To determine the regulatory elements in the FMR1 IRES, C6 cells were exposed to 500 μM ameloride for 24 hrs to block the Na+/H+ antiporter. Intracellular acidification inhibits neural activity and is a model of an inactive neuron [35]. The P:R ratio from the dicistronic mRNA containing the full-length 5' leader dramatically decreased in the presence of ameloride (Fig. 4A). However, only subtle differences in the P:R ratio were observed from truncations of the 3' 208 nt. The minimal level of IRES activity remained in the shorter leaders. This result implies that a decrease in intracellular pH inhibits FMR1 IRES activity and this effect is mediated by repressing the IRES-promoting region located at the 5' end of the 5' leader.

### The CGG repeats contribute to FMR1 IRES activity

The basal level of IRES activity exhibited in the 5' leader containing the 3' 120 nt was not affected by changes in intracellular pH. An additional truncation deleting the CGG repeats abolished all IRES activity. To further characterize the role of the CGG repeats in the FMR1 IRES, the repeats were internally deleted within the full-length 5' leader. Transfection of the dicistronic mRNA containing the FMR1 5' leader with the CGG repeats deleted exhibited an approximately 50% decrease in the P:R ratio compared to the full-length 5' leader (Fig. 4B). This result demonstrates the importance of the CGG repeats for internal initiation mediated by the FMR1 5' leader.

### FMR1 IRES activity is affected by KCl and polyinosinic:polycytidylic acid

**Figure 5:**
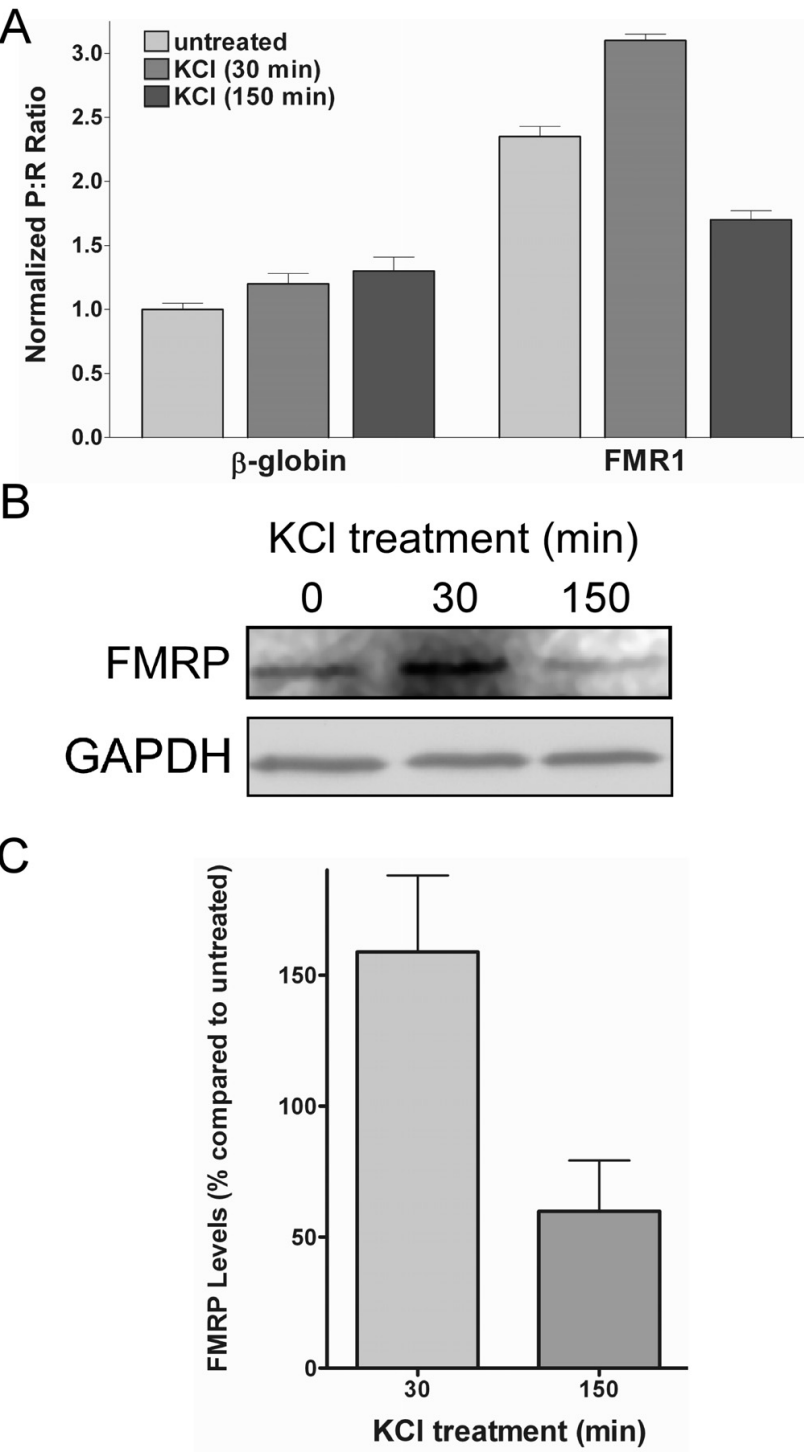
**Exposure to KCl alters FMR1 IRES activity and FMRP expression.** (A) Dicistronic luciferase mRNA containing the β-globin or FMR1 5' leader inserted into the intercistronic region was transfected into C6 cells and exposed to 50 mM KCl for 30 or 150 min. After seven hr the cells were assayed for *Photinus *and *Renilla *luciferase activity. (B) Lysates from cells treated in (A) were analyzed for FMRP and GAPDH (as a loading control) using Western blots. Shown is a representative Western blot; each experiment was performed in triplicate, n = 3. (C) Protein expression was quantitated using ImageQuant software; FMRP levels were normalized to GAPDH expression.

IRES-dependent translation is affected by multiple cellular stimuli [36]. We sought to determine whether the FMR1 IRES is regulated by environmental stimuli which regulate the processes in which FMRP participates. Neural activity leads to translation of FMR1 mRNA [2,37] and we modeled this phenomenon by treating cells with 50 mM KCl for 30 or 150 min. The short KCl exposure led to a 32% increase in the P:R ratio from the dicistronic mRNA containing the FMR1 5' leader (Fig. 5A). However, the longer KCl treatment resulted in a 28% decrease in the P:R ratio. Moreover, the FMR1 P:R ratio after the 150 min treatment was only one-third higher than that obtained from the control dicistronic mRNA. This result indicates that cellular depolarization differentially affects FMR1 IRES activity depending upon its duration.

To examine whether KCl also affects FMRP expression, Western blots were performed from lysates obtained from the treated and untreated cells. Changes in endogenous FMRP levels mirrored that observed from FMRP IRES (Fig. 5B, C). An increase of 58% was seen after a 30 min KCl treatment, whereas a 150 min treatment led to a 40% decrease in FMRP expression.

**Figure 6:**
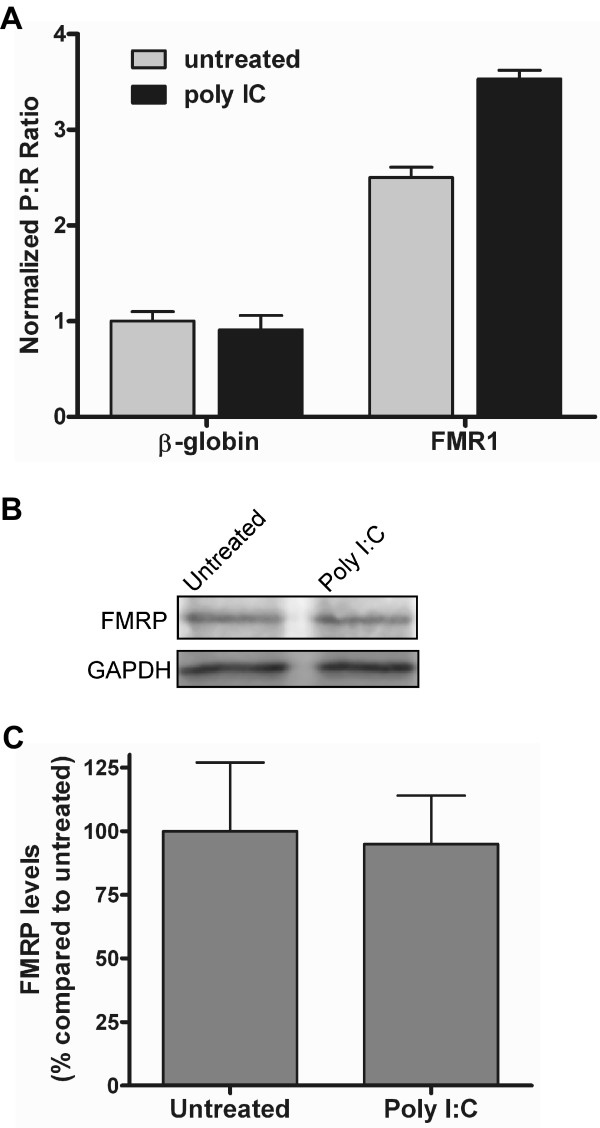
**Exposure to poly I:C alters FMR1 IRES activity.** (A) Dicistronic luciferase mRNA containing the β-globin or FMR1 5' leader inserted into the intercistronic region were transfected into C6 cells and exposed to 500 μg/ml of poly I:C. After seven hr the cells were assayed for *Photinus *and *Renilla *luciferase activity. (B) Lysates from cells treated in (A) were analyzed for FMRP and GAPDH (as a loading control) using Western blots. Shown is a representative Western blot; each experiment was performed in triplicate, n = 3. (C) Protein expression was quantitated using ImageQuant software; FMRP levels were normalized to GAPDH expression.

FMRP has been localized to the RNA-induced silencing complex (RISC), a nuclease complex that mediates RNA interference (RNAi). Deletion of FMRP leads to a loss of RNAi [38]. To determine if the presence of double stranded RNA affects FMR1 IRES activity, C6 cells were exposed to 500 μg/ml of polyinosinic:polycytidylic acid (poly I:C), a double stranded polyribonucleotide. After a 7 hr exposure to poly I:C., the FMR1 P:R ratio increased by 41% (Fig. 6A). This result indicates a possible positive feedback mechanism to stimulate FMRP synthesis in response to double stranded RNA and it predicts that RNAi activity will increase FMR1 translation in an IRES-dependent manner. Tempering this conclusion was that expression of endogenous FMRP did not change after a 7 hr exposure to poly I:C (Fig. 6B, C). As was observed from the KCl experiments, it is possible that FMRP expression may be upregulated at different timepoints following poly I:C exposure.

## Discussion

In the present report we demonstrate that the FMR1 5' leader contains an IRES whose activity is dependent upon the 5' 45 nt as well as the CGG repeats in the 5' leader. Moreover, our studies indicate that internal initiation may be an important mechanism for the translation of the FMR1 mRNA. This conclusion is supported by the observation that synthesis of FMRP is maintained when cap-dependent translation is inhibited by knocking-down eIF-4E expression or rapamycin treatment. In addition, multiple stimuli differentially affect FMR1 IRES activity suggesting that similar processes are dynamically regulating FMR1 translation in the cell.

Our results show that the FMR1 5' leader contains a cryptic promoter, an observation that has been noted in other 5' leaders [31,39]. The presence of elements influencing transcription is not surprising as transcriptional elements are located throughout a gene. While the FMR1 5' leader affects transcription when present as DNA, the same region promotes internal initiation as RNA as deduced from both *in vitro *and *ex vivo *experiments using monocistronic and dicistronic mRNA. RNA for these assays was produced from *in vitro *transcription and this process could yield a FMR1 5' leader with a secondary structure different than what occurs in the cell. Indeed, it is likely that proteins including IRES transactivating factors (ITAFs, see below) bind to the FMR1 5' leader and alter its structure *in vivo*. However, viral IRESes whose activity depends extensively upon secondary structure yield robust IRES activity from *in vitro *transcribed RNA [40,41] and are suitable for structural analysis [42].

The CGG repeats, which are amplified in FXS, are evolutionarily conserved in mammals [18]. We suggest that the CGG repeats are retained due to their ability to promote translation and specifically internal initiation of translation. This hypothesis is supported by evidence indicating that mRNA containing the normal number of CGG repeats translates at a higher level compared to mRNA absent of the repeats or containing a higher number of repeats [43]. Moreover, we found that deleting the CGG repeats significantly decreased FMR1 IRES activity. The mechanism by which the repeats affect IRES activity is open to speculation. Secondary structure is important for viral IRESes and their ability to recruit canonical factors and the ribosome [44]. Minor changes in the RNA structure can dramatically alter viral IRES activity [45,46]. Since the 3' 120 nt are able to mediate internal initiation, the ribosome must bind somewhere in this region and the CGG repeats may create a structure conducive for ribosomal recruitment. On the other hand, it has been proposed that expansion of the CGG repeats (~50 – 200) in the Fragile X pre-mutation allele sequesters an RNA binding protein and indirectly affects the function of other mRNAs [47]. Thus, the CGG repeats may bind an ITAF that directly recruits the translational machinery or may act as a molecular chaperone and alter the secondary structure, which in turn recruits the translational machinery.

Deletion of the 5' 45 nt (or more specifically the nt 18 – 45 from the 5' end) of the FMR1 5' leader yielded the largest decrease in IRES activity. This segment is also very conserved in mammals. In general, viral IRESes are greater than 200 nt in length and it would be of interest if a substantially smaller RNA segment of the FMR1 5' leader could internally initiate translation. This observation is not unprecedented as we have found that a region of 50 nt in the 5' leader of the amyloid precursor protein yields IRES activity (Beaudoin et al. submitted). However, the 5' deletion analysis as discussed above indicates that the ribosome binds further downstream. It is likely that the 5' 45 nt does not contain an IRES, but acts as an enhancer by affecting downstream RNA secondary structure, perhaps through protein binding. Of interest is a region of ten contiguous nt (nt 33 – 42 from the 5' end) of which nine are pyrimidines making this a potential binding site for the polypyrimidine binding protein PTB) and its neural homolog nPTB. PTB aside from its role in RNA splicing is an important ITAF for many eukaryotic IRESes [48].

FMRP is synthesized in response to neural activity [2,37] and in particular, a brief exposure to KCl stimulates FMR1 synthesis in neuronal dendrites [49]. In our study, both internal initiation mediated by the FMR1 5' leader and FMRP expression were also regulated by the duration of KCl exposure; a short exposure increased and a longer exposure decreased FMR1 IRES activity and FMRP expression. Moreover, intracellular acidification associated with decreased neural activity also inhibited FMR1 IRES activity. These results suggest that neural activity of differing intensity or duration may produce distinct changes in IRES-dependent translation mediated by the FMR1 5' leader and that IRES-dependent translation is a mechanism contributing to the synthesis of FMRP in neurons. These results also indicate that a feedback mechanism exists that is dependent upon the duration of the calcium influx through voltage gated calcium channels stimulated by KCl. Indeed, extent and duration of intracellular calcium can regulate the translation of other mRNAs [50,51].

FMRP is associated with the RISC complex [7,8] and our results indicate that the presence of double stranded RNA stimulates IRES-dependent synthesis of FMRP. This result implicates a positive feedback mechanism regulating the synthesis of FMRP and the level of FMRP could regulate the activity or the targets of the RISC complex. However, the overall level of FMRP was not altered after poly I:C exposure. Poly I:C leads to phosphorylation of eIF-2α [52] and a subsequent decrease in cap-dependent translation [53]. It is possible that the loss of any cap-dependent translation of the FMR1 mRNA is compensated by an increase in IRES-dependent translation, but the global level of FMRP is unaffected.

## Conclusion

In summary, we demonstrated that the FMR1 5' leader contains an IRES, whose critical elements include the CGG repeats as well as the 5' end of the leader. Inhibiting cap-dependent translation *in vitro *and *ex vivo *resulted in only a small diminution in the translation of reporter mRNAs containing the FMR1 5' leader. Under similar conditions, endogenous FMRP expression was maintained. Multiple stimuli altered internal initiation of translation mediated by the FMR1 5' leader that in some cases mirrors that of FMRP synthesis *in vivo*. Taken together, our study indicates that IRES-dependent translation of the FMR1 mRNA may be a major contributor to the synthesis of FMRP *in vivo*.

## Methods

### Constructs

The FMR1 5' leader was PCR amplified from a human brain cDNA library (Clontech) and inserted into the dual luciferase vector – pRF [54,55] (a generous gift from Dr. Anne Willis, University of Leicester) with EcoRI and NcoI restriction endonuclease sites. The promoterless construct was created by digesting the dicistronic construct with SmaI and EcoRV and religating the construct. The monocistronic vector for the *ex vivo *experiments was created by digesting the RP vector with EcoRI and BamHI. The digest released the 5' leader, the *Photinus *luciferase gene, and the SV40 3' UTR, which were cloned into the pGL3 vector (Promega). The monocistronic vector for the *in vitro *experiments was created by inserting the above *Photinus *gene into the SK+ Bluescript vector (Stratagene) downstream of a T7 promoter.

Serial truncations were produced by PCR amplification with 5' and 3' primers containing EcoRI and NcoI endonuclease restriction sites, respectively. Deletion of the CGG repeats was accomplished by amplifying the region 3' to the repeats in the FMR1 5' leader and inserting it into the pRF construct with EcoRI and NcoI restriction sites on the 5' and 3' end, respectively. The 5' leader upstream of the CGG repeats was amplified and inserted upstream of the 3' FMR1 5' leader using EcoRI restriction sites. In experiments using a hypophosphorylated form of 4E-BP1 (containing Thr-37-Ala/Thr-46-Ala mutations), plasmids expressing this protein or the parent vector (both based on pACTAG-2) were co-transfected with the monocistronic constructs described above, using a 8-fold molar excess of the 4E-BP1 or control expression constructs [20]. The 4E-BP1 double mutant and control expression plasmids were generously provided by Dr. Nahum Sonenberg (McGill University, Montreal).

### *In Vitro *Translation

The Bluescript SK+ vector containing the 5' leaders upstream of the *Photinus *luciferase gene was linearized with BamHI and *in vitro *transcribed using mMessage Machine (Ambion) producing capped mRNA. The mRNA was extracted with phenol/chloroform and a sample was run on an agarose gel to ensure RNA integrity. 0.5 μg of the mRNA and 1.6 nM methionine was added to rabbit reticulocyte lysate (Speed Read, Novagen) and incubated for 1 hr at 30°C in the presence or absence of cap analog (Ambion). The sample was subsequently assayed for *Photinus *luciferase activity.

### Cell Culture/Luciferase Assays

C6 cells were obtained from ATCC and cultured in DMEM, 10% fetal bovine serum and 2 mM L-glutamine. Polyinosinic:polycytidylic acid, ameloride, and KCl were obtained from Sigma. Cells were transfected with 2 μg of DNA using Fugene transfection reagent (Roche) or 4 μg of mRNA using the TransMessenger RNA transfection reagent (Qiagen) according to the manufacturer's directions. The RNA was *in vitro *transcribed using the mMessage Machine (Ambion) and purified as detailed above. After 24 hr (DNA transfections) or 7 hours (RNA transfections), the cells were lysed with 500 μl of lysis buffer (Promega). Forty μl of the supernatant were used for the luciferase assays using the Dual-Luciferase Reporter Assay System and analyzed in a Luminoskan luminometer. For the siRNA experiments, C6 cells were transfected with 10 pmol of rat siRNA targeted against eIF-4E (On-Target plus SMARTpool, cat# L-0088826-01, Dharmacon) or nonsense siRNA (Dharmacon) using INTERFERin siRNA transfection reagent (Polyplus-Transfection) as directed by the manufacturer. Cells were lysed with 200 μl of lysis buffer (Promega) including phosphatase (Pierce) and protease (Roche) inhibitors after 72 hours. The cell lysates were analyzed by Western blot.

### Western blot analysis

Cells were harvested in cell lysis buffer (Promega) with protease (Roche) and phosphatase inhibitors (Pierce). The cell lysate was analyzed by Western blot by separating the proteins on a 12% SDS-polyacrylamide gel and transferred onto nitrocellulose. The membranes were blocked (Invitrogen) and probed with a monoclonal antibody (7G-1, 1:1000 dilution; Developmental Hybridoma Bank) or a polyclonal antibody (sc-28739, 1:200 dilution; Santa Cruz) directed against FMRP. Both antibodies mainly recognized a single band at approximately 80 kD in lysates from C6 cells and rat hippocampus (see Additional file 1). In addition monoclonal antibodies directed against phosphorylated p70 S6 kinase (9206,1:1000 dilution; Cell Signaling) and eIF-4E (610269; 1:500 dilution, BD Biosciences) or polyclonal antibodies directed against JunB (Ab31421, 1:500 dilution; Abcam), and GAPDH (sc-25778, 1:200 dilution; Santa Cruz) were used in 5% nonfat dried milk in a solution of PBS containing 0.1% Tween 20. The blots were then incubated with alkaline phosphatase-conjugated secondary antibodies (1:10,000 – 1:50,000 dilution; Invitrogen) for 1 hour. Immunoreactive bands were detected using chemiluminescence (Invitrogen) as per manufacturer's directions. Westerns blots were either from individual PAGE gels (Fig. 3A) or the nitrocellulose was restripped and reprobed (Figs. 3B, 5B, 6B). The Western blots were quantitated using ImageQuant software (Applied Biosystems).

## List of Abbreviations

The abbreviations used are: FMR1: FMR1 Fragile X gene; IRES: internal ribosomal entry site; 4E-BP1: 4E-Binding Protein 1; EMCV: encephalomyocarditis virus; FMRP: FMR1 protein; mTOR: mammalian target of rapamycin; siRNA: short interfering RNA; poly I:C: polyinosinic:polycytidylic acid; P:R: *Photinus*: *Renilla *luciferase; hr: hour; min: minute.

## Authors' contributions

TD contributed to all of the methodological procedures and aided in the design of the study and drafting of the manuscript. EK generated constructs, performed cellular transfections and the subsequent luciferase assays. ST cloned the wild-type FMR1 gene and performed the *in vitro *translation experiment. LAK generated constructs, designed the study, and drafted the manuscript.

## Supplementary Material

Additional file 1 **Different FMRP antibodies recognize similar proteins in hippocampal extract and C6 cells.** Western blots of lysates from rat brain hippocampus and C6 cells were probed with a mouse monoclonal (7G1, Developmental Hybridoma Bank) and a rabbit polyclonal (sc-28739, Santa Cruz Biotechnology) antibody, both of which are directed against FMRP. The two antibodies mainly recognize a single band of approximately 80 kD. Click here for file
