# Supplementary figures and images for: Identifying intrinsic and extrinsic determinants that regulate internal initiation of translation mediated by the FMR1 5' leader
Source: BMC Mol Biol. 2008 Oct 15;9:89. doi: 10.1186/1471-2199-9-89 (PMC2576346; doi:10.1186/1471-2199-9-89)

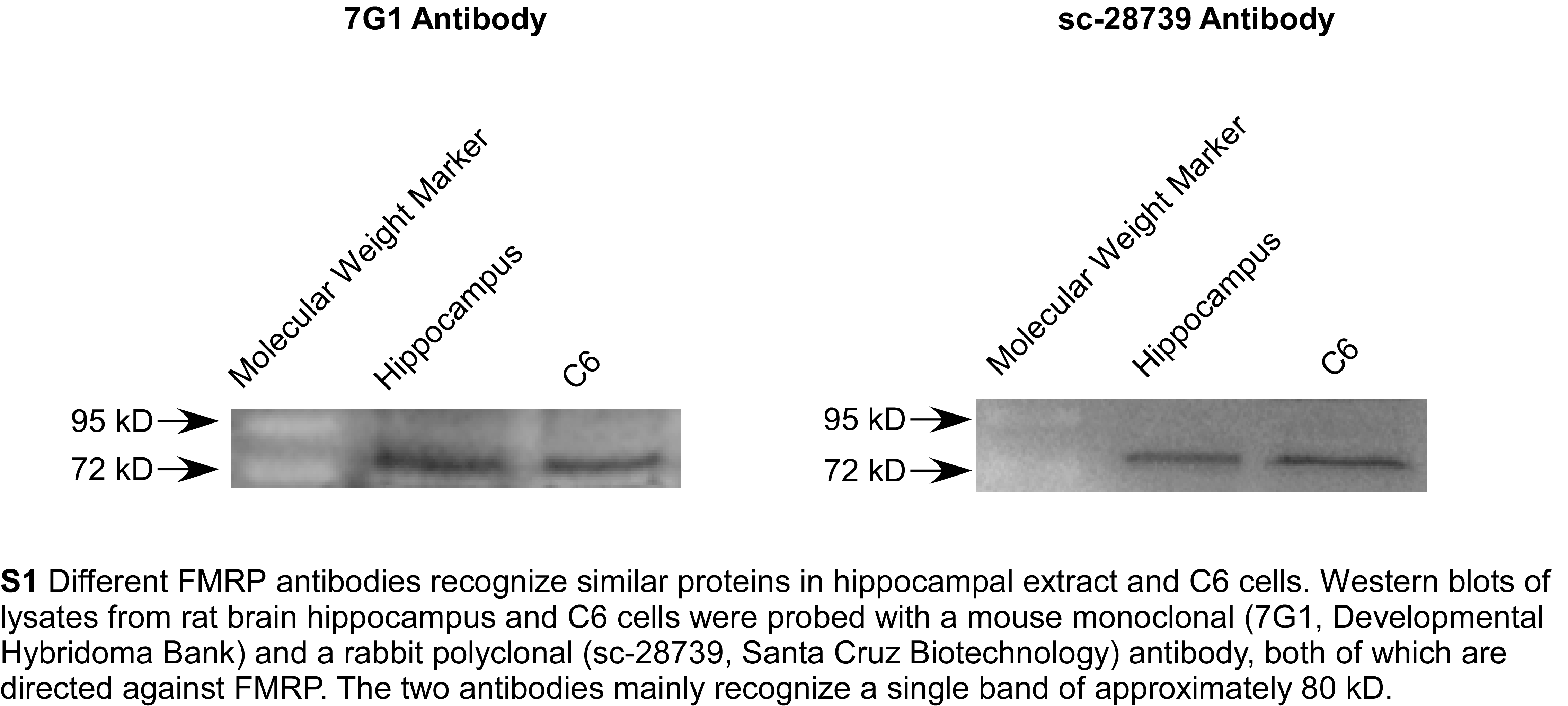

Supplement: Additional file 1 — Different FMRP antibodies recognize similar proteins in hippocampal extract and C6 cells. Western blots of lysates from rat brain hippocampus and C6 cells were probed with a mouse monoclonal (7G1, Developmental Hybridoma Bank) and a rabbit polyclonal (sc-28739, Santa Cruz Biotechnology) antibody, both of which are directed against FMRP. The two antibodies mainly recognize a single band of approximately 80 kD. [file 1471-2199-9-89-S1.tiff]
